# Unilateral cerebellar hypoplasia in a 9-year-old child with chronic granulomatous disease: A case report

**DOI:** 10.1016/j.radcr.2023.08.009

**Published:** 2023-08-26

**Authors:** Ali Hajihashemi, Mahsa Geravandi

**Affiliations:** Department of Radiology, Isfahan University of Medical Sciences, Hezar Jerib Ave, Isfahan, Iran

**Keywords:** Unilateral cerebellar hypoplasia, Cerebellar malformation, Chronic granulomatous disease

## Abstract

Unilateral cerebellar hypoplasia is a rare neurological condition that affects the development of the cerebellum, causing symptoms like poor coordination, balance issues, tremors, and speech problems. Unilateral cerebellar hypoplasia can occur as an isolated finding or as part of a larger neurological disorder or hereditary disease. There have been rare recorded instances where patients with chronic granulomatosis disease have been found to have neurological symptoms, such as brain abscesses or persistent inflammation, even though that CGD primarily affects the immune system and causes recurrent infections.

A 9-year-old male with a known diagnosis of CGD presented to our neurology outpatient department with complaints of frequent falls and speech abnormalities. His parents described suspicious seizure-like movements and poor scholarly performance. Neurologic examination showed ataxic gait, slurred speech, and right-sided plantar extensor reflex. Initial laboratory findings were normal. MRI revealed marked reduced volume of the left cerebellar hemisphere with intact vermis and asymmetry of the posterior fossa. The residual left cerebellar hemisphere showed a normal folia and gray-white matter differentiation pattern. CSF filled the space created by the left hypoplastic cerebellum. A diagnosis of unilateral cerebellar hypoplasia was made.

There is no known direct association between chronic granulomatous disease and unilateral cerebellar hypoplasia. However, more research is required to discover whether there is any connection between them. Although it is possible for a child to have CGD and UCH, managing such cases requires a multidisciplinary approach involving neurologists, immunologists, and other specialists to provide appropriate care and treatment.

## Background

Unilateral cerebellar hypoplasia (UCH) is a rare condition where one of the cerebellum hemispheres is underdeveloped [1]. It can be diagnosed prenatally or later in life and associated with other developmental abnormalities [1,2]. The frequency of cerebellar hypoplasia as a congenital neurological condition is unknown [3]. Symptoms can include seizures, developmental delays, and difficulty with coordination and balance. However, some patients may be asymptomatic. Clinical findings may vary depending on the condition's severity and the patient's age [4]. Diagnosis is typically made through imaging, such as magnetic resonance imaging (MRI). Treatment may involve managing symptoms and providing support for developmental delays [5].

## Case presentation

A 9-year-old male with a known diagnosis of chronic granulomatous disease (CGD) presented to our neurology outpatient department with complaints of frequent falls and speech abnormalities. He had trouble walking and speech difficulties characterized by slurred speech and nasal intonation. There was no significant medical history of meningoencephalitis, febrile seizures, or head trauma. No history of recent persistent headaches or delays in development was described. His parents described suspicious seizure-like movements without fever twice during childhood, but no further evaluation was performed. They told the history of his poor scholarly performance. The birth history revealed he had been admitted to the intensive care unit because of low birth weight and respiratory distress. There was no evidence of known consanguinity in family history.

A general examination showed no dysmorphic features. Cranial nerve examination demonstrated no pathology. The motor examination was normal. Neurologic examination showed ataxic gait, slurred speech, and right-sided plantar extensor reflex with accentuated deep tendon reflexes. Other system examinations revealed no abnormalities.

**Fig. 1:**
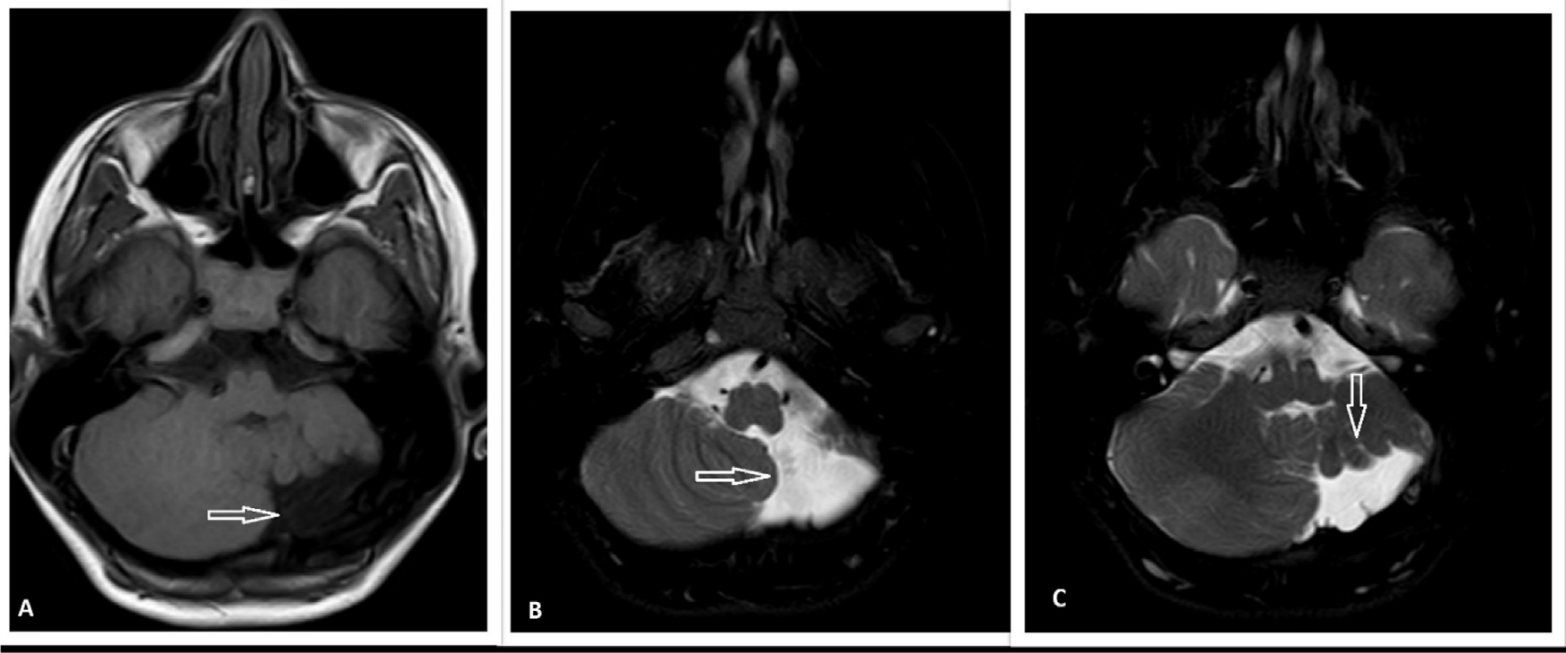
Axial T1W (A), T2W (B, C) sequences brain MRI of left cerebellar hypoplasia. There is marked hypoplasia of the left cerebellar hemisphere (arrows) and normal volume of the right cerebellum. No signal abnormality or gliosis in the left cerebellum is seen. The right cerebellar hemisphere is normal in bulk and signal intensity. The normal bulk of the vermis and brain stem can also be seen.

**Fig. 2:**
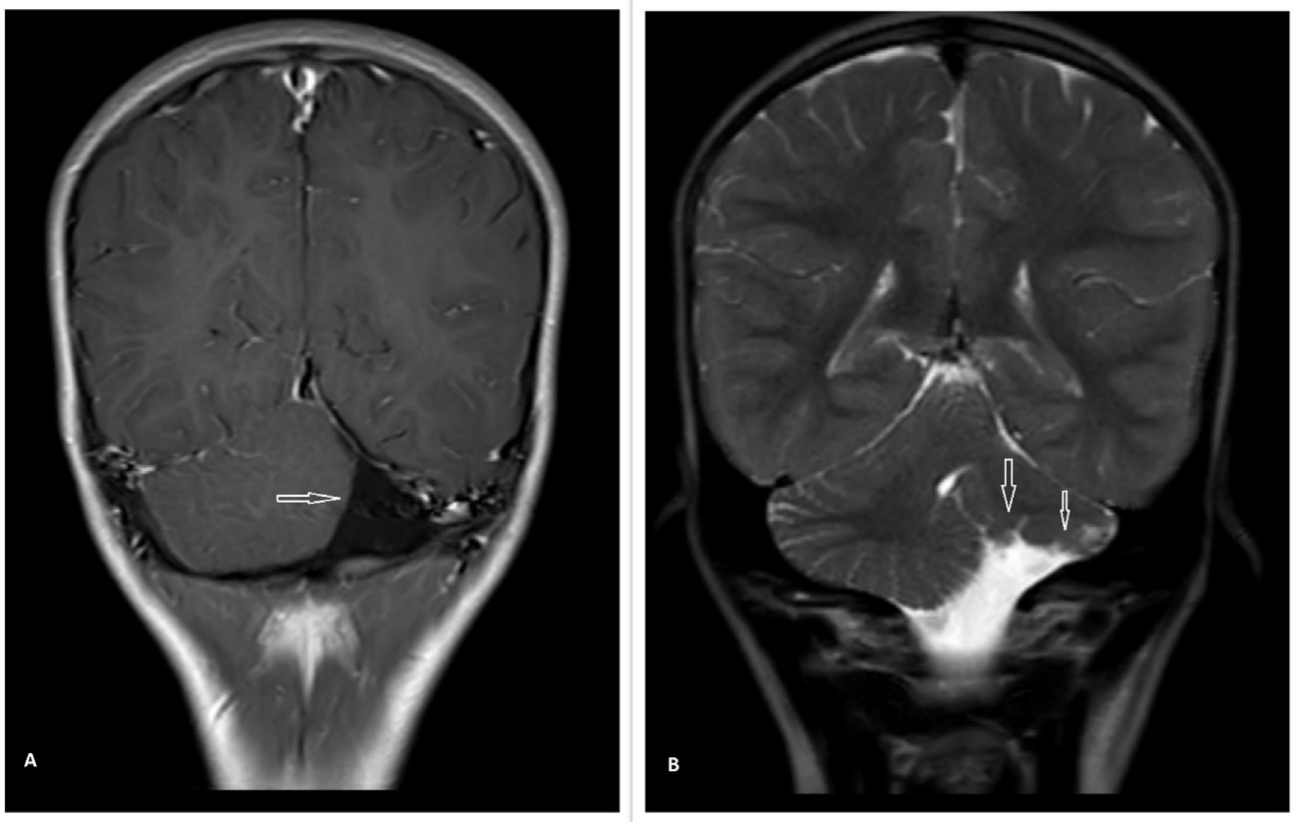
Coronal T1W with GAD (A), T2W (B) sequences brain MRI of left cerebellar hypoplasia. The residual left cerebellum shows normal gray-white matter differentiation and foliation (arrows). The right cerebellar hemisphere shows normal volume and morphology.

Initial laboratory findings were normal. An electroencephalogram (EEG) was done because of suspicious seizure-like movements, which were normal. Multiplanar and multisequence contrast MRI of the brain was done. It revealed a markedly reduced volume of the left cerebellar hemisphere with intact vermis and asymmetry of the posterior fossa, with the left side more diminutive than the right (Fig. 1). The residual left cerebellar hemisphere showed normal folia and a gray-white matter differentiation pattern. No evidence of signal change or gliosis was detected. Cerebrospinal fluid leak (CSF) filled the space created by the left hypoplastic cerebellum. The right cerebellar hemisphere was normal in bulk and signal intensity (Figs. 1 and 2). The midbrain, medulla, pons, fourth ventricles, and cerebral hemispheres were also normal. A UCH diagnosis was made in light of these MRI results. Rehabilitation and therapy were considered for improving motor function and the quality of his life.

## Discussion

UCH is a rare condition where one of the cerebellum hemispheres is underdeveloped [1]. It can be diagnosed prenatally or later in life and associated with other developmental abnormalities [1,2]. The frequency of cerebellar hypoplasia as a congenital neurological condition is unknown. Patel and Barkovich [6] proposed a rational classification of cerebellar abnormalities in 2002 with precise descriptions and usable criteria. Based on imaging criteria, cerebellar malformations are divided into those with hypoplasia and those with dysplasia. Hypoplasia is a term used to describe a decreased cerebellar volume with normal cerebellar shape and texture or normal folia and fissure size and pattern. Dysplasia is the abnormal pattern of foliation and/or gray matter heterotopia.

UCH commonly affects one side of the cerebellum, with or without the vermis, which can lead to asymmetrical motor function and coordination issues. Numerous variables, including genetic mutations, infections, and environmental conditions, might contribute to the condition. Also, it is thought to result from a vascular (hemorrhagic or ischemic) injury during pregnancy. UCH may have no recognized cause [2].

Depending on the severity of the disorder and the specific cerebellum region affected, UCH symptoms can vary widely. The following are some typical signs of UCH:

• Clumsiness and issues with coordination and balance
• Delayed motor growth, including walking and crawling
• Jerky or trembling movements
• Having trouble with fine motor activities like writing or using tools
• Speech and language issues, such as difficulty pronouncing words or slurred speech
• Vision issues like nystagmus (uncontrollable eye movements)
• Delays in development or intellectual disabilities can cause cognitive and learning problems.
• Despite being less frequent, neurological disorders include epilepsy [4,5,[7], [8]–9].

Depending on the patient's symptoms and medical history, various methods of diagnosis may be performed.

On the other hand, the immune system is the main organ affected by CGD, which frequently results in infections and inflammation. The CNS can be involved in CGD through infection and inflammation. It can cause meningitis, encephalitis, or abscesses in the brain, delays in cognitive and developmental milestones due to the effects of recurrent infections, and also vasculitis [10]. In our case, it can cause sequelae in the long term, such as parenchymal brain atrophy, parenchymal gliosis, and signal changes.

In this situation, evaluating and monitoring patients with CGD for any potential long-term effects following brain inflammation or infection is important. Currently, there is no evidence indicating a correlation between CGD and UCH. However, additional research is necessary to determine whether they have a relationship.

### Declaration of generative AI and AI-assisted technologies in the writing process

During the preparation of this work, the author(s) used [Grammarly] in order to [ grammar check, improving the clarity, style, and tone of your writing]. After using this tool/service, the author(s) reviewed and edited the content as needed and take(s) full responsibility for the content of the publication.

## Patient consent

Consent was obtained.
